# Acute Confusional Migraine: Distinct Clinical Entity or Spectrum of Migraine Biology?

**DOI:** 10.3390/brainsci8020029

**Published:** 2018-02-07

**Authors:** Ashar M. Farooqi, Jennifer M. Padilla, Teshamae S. Monteith

**Affiliations:** Department of Neurology-Headache Division, University of Miami, Miller School of Medicine, Miami, FL 33136, USA; farooqam@ucmail.uc.edu (A.M.F.); Jennifer.Padilla1@BSWhealth.org (J.M.P.)

**Keywords:** acute confusional migraine, aura, disorientation, agitation, International Classification of Headache Disoders-3 beta version, migraine variant, cognitive, diagnosis

## Abstract

The goal of this review is to explore the literature reports of acute confusional migraine (ACM) including patient characteristics, migraine symptomatology, and proposed diagnostic criteria. A literature review was conducted using PubMed, Scopus and Web of Science using the terms “confusional migraine” and “confusional state in migraine”. All the relevant articles from 1970 to 2016 were included. A total of 120 patients were found in the literature. Most of the cases were seen in the pediatric population with a slight male predominance. Personal or family history of migraine was common. Most patients had a headache prior to the confusional state. In addition to confusion and agitation, some developed visual (32.5%) and/or sensory symptoms (19%) and/or speech problems (39%) either prior to or during the confusional state. Data on treatment outcomes is lacking. Patients with most common forms of migraine report attention and cognitive disturbances but awareness remains intact as opposed to patients with ACM. ACM is a distinct entity and should be included as part of the appendix of International Classification of Headache Disoders-3 beta version (ICHD-3β) criteria. Prospective studies are needed to further study this disorder and its association with other migraine forms.

## 1. Introduction

Migraine is best thought of as a multiphasic brain disorder, often with a premonitory and postdrome phase associated with the headache. The diagnosis is characterized by moderate to severe pain intensity, usually unilateral, lasting from 4–72 h, in addition to other associated symptoms such as nausea, vomiting, sensitivity to light, sound, and movement. Some patients have transient neurological disturbances called auras. Auras can cause visual, language, motor and sensory disturbances as well as brainstem symptoms as dysarthria, vertigo, tinnitus, ataxia, double vision, and decreased loss of consciousness. Migraine is a common type of headache in the pediatric population, but the attacks can differ from those in the adult population. According to the International Headache Society’s International Classification of Headache Disoders-3 beta version (ICHD-3β), recurrent gastrointestinal disturbance, benign paroxysmal vertigo, and benign paroxysmal torticollis are episodic syndromes that may be associated with migraine and are historically noted to occur in childhood, although they occur less frequently in adults. Moreover, a state of confusion during a migraine attack was first described by Gascon and Barlow, in 1970, in four children aged from 8–16 years old [1]. In 1978, Ehyai and Fenichel named this migraine variant as acute confusional migraine (ACM) [2]. Although not a part of the ICHD-3β, ACM is an important clinical entity that often causes high levels of disability, diagnostic uncertainty, and may be the first presentation of a migraine in children and/or adolescents.

ACM is a migraine variant that manifests with acute confusion, agitation, disorientation, altered mental status, speech difficulties and memory deficits [1,2,3]. Several classification criteria have been proposed. There is a paucity of related literature and limited awareness, which may be in part due to its exclusion from the ICHD-3β. The primary objectives of this review are to summarize all the cases of ACM reported to date and evaluate for evidence that supports ACM as a distinct clinical entity versus a manifestation of migraine biology based on migraine phases.

## 2. Materials and Methods

**Figure 1:**
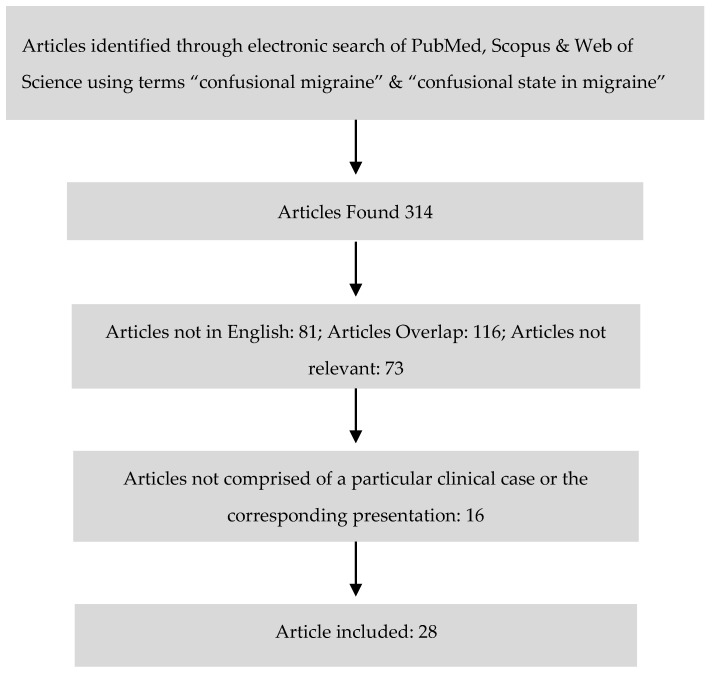
Electronic Literature Search for ACM Cases.

A literature review was done using PubMed, Scopus and Web of Science using the term “confusional migraine” and “confusional state in migraine” (Figure 1). The articles that were not written in English language and did not entail and encompass clinical case presentation of ACM were excluded. One abstract that was presented at various scientific meetings and conferences during poster session was also not included, as it did not contain corresponding information about clinical presentation in detail. The articles related to confusional states in children and adult attributed to medical conditions other than migraine were not included. All the relevant articles, case reports and series, published and presented in different meetings from 1970 to 2016 were included as shown in the flow chart.

There are 28 case reports and series related to ACM in the literature with a total number of 120 patients. (Table 1).

## 3. Results

### Clinical Features

*Prevalence*: The prevalence of these migraine variants varies in literature. In a study that included 5848 patients, of whom 1106 had migraine, it was noted that 9.8% of those with migraine had migraine equivalents. Among different types of migraine equivalents, about 5% were identified as having ACM [16]. In another study, of 2509 patients, aged 0–18 years old, migraine variants were seen in 24% of those with migraine and 2.7% of these were reported as having ACM [26].

**Figure 2:**
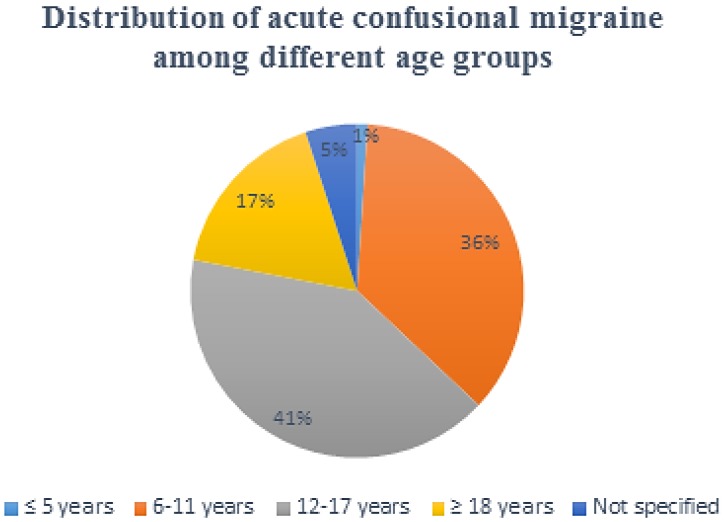
Schematic distribution of acute confusional migraine among different age groups. The figure shows two-thirds (75%) of the cases occur in people aged <18 years.

*Age and Gender*: ACM was reported mostly in the pediatric population, and less commonly in adults. About three fourth of these cases were seen in the children and adolescents (5–17 years) as shown in Figure 2. Only a few case series included patients who were older than 18 years old [6,20,23,27,29]. A slight male predominance (57%) is evident among all of the reported cases (68 males/52 females).

*Past medical and family history*: Relevant personal or family history of migraine was present in the majority of cases. Among the ACM cases reported to date, a personal and family history of either prior migraine or headache was present in 54% and 62% patients, respectively. A history of mild head trauma without loss of consciousness prior to the confusional state was present in 33 patients (27%), absent in 50 patients (42%), and was not reported in 37 patients (31%).

**Figure 3:**
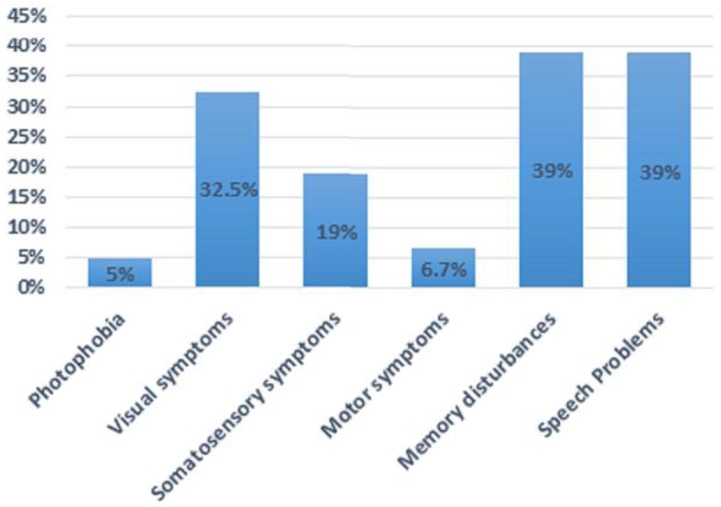
Percentage of different symptoms reported by the patients with ACM. In addition to confusion, common symptoms include visual, sensory, motor, memory and speech disturbances.

*Clinical presentation*: Confusion, disorientation and agitation are the cardinal and distinctive features, followed or accompanied by headache. About 69 patients (58%) complained of headache prior to the confusional state, and 11 patients (9%) developed headache after the confusion was resolved. Although headache was present in the remaining patients, the time of onset was unspecified because either the patients were confused or amnesia was present during the episode. In addition to confusion, transient visual and somatosensory symptoms including sudden blindness, scotoma, numbness or paresthesia along with speech and memory disturbances were also reported (Figure 3). These neurological changes were mild and usually resolved within 24 h. The confusion duration was variable and in some of the cases was not reported. It ranged from 15 min to 72 h, although most lasted less than 24 h. By the end of attack, patients became drowsy and fell to sleep. The confusion and accompanying symptoms ceased spontaneously after deep sleep in 19 patients (16%); however, it was not mentioned in the remaining cases. After resolution, patients usually had partial or global amnesia of the episode.

*Diagnostic testing*: Routine laboratory work performed in the majority of patients were reported as normal. Cerebrospinal studies (CSF) analyses were performed in some of the cases and did not show abnormalities in opening pressure, CSF chemistry or cytology. Magnetic resonance imaging (MRI) performed during the episodes of confusion was completely normal. However, single-photon emission computerized tomography (SPECT) scans showed decreased cerebral blood flow in the left splenium region [13], and medial temporal lobe [21]. Magnetic resonance imaging (MRA) performed during confusion in a patient with a history of recurrent ACM revealed reversible narrowing of left middle and posterior cerebral arteries [19]. EEG recording during attack showed diffuse delta slowing more prominent on the occipital region of dominant hemisphere and at times frontal intermittent rhythmic delta activity (FIRDA) pattern. No significant abnormalities were observed in post-ictal polysomnograph, except for some non-specific changes during nocturnal awakenings [4]. A gradual improvement of EEG abnormalities and recovery of physiologic rhythms occurred once the confusion and altered sensorium was cleared [6]. EEG findings can help in differentiating ACM from transient global amnesia (TGA), as EEG in the latter is usually normal. Ictal EEG recordings suggest transient neuronal dysfunction at the subcortical and upper brain stem level [7].

*Treatment*: No specific therapy was mentioned in most case reports; however, some reports suggested efficacy of sodium valproate and prochloroperazine as treatment modalities for acute confusional state [12,19,21,22]. Prophylactic treatment with topiramate seems to be effective in the treatment of recurrent ACM [24]. Recently, propofol was given to a patient who developed confusion and agitation following headache, to achieve sedation. However, after a brief period of time, his consciousness improved, and no further confusion was reported the following day [29].

*Outcomes*: The longitudinal course of ACM is unknown, mostly due to anecdotal reports. In a few cases, recurrence of similar confusional episodes has been documented. Children, in whom confusional state was the initial presentation, developed migraine with or without aura over a period of time. In one study, among the adults, ACM was noted to be an early presentation of cerebral autosomal dominant arteriopathy with subcortical infarcts and leukoencephalopathy (CADASIL) [21].

## 4. Discussion

ACM manifests with acute confusion, agitation, disorientation, speech difficulties and memory deficits. It is most commonly seen in children and adolescents and less commonly seen in adults. It is considered a migraine variant, which is defined as an episodic syndrome that occurs in patients with migraine with or without aura or in those who have a tendency towards developing them [30]. ACM is a diagnosis of exclusion and therefore other potential etiologies such as infection, seizures, inflammatory, neoplastic, transient global amnesia, vascular and metabolic abnormalities should be ruled out first. In addition, migraine is comorbid with a wide range of other medical conditions such as cerebrovascular disease, epilepsy, and metabolic disorders. Similar to what has been referred to as late-life migraine accompaniments, ACM can be a cause of unexplained transient neurological events [31]. Moreover, both migraine and epilepsy are characterized by paroxysmal brain dysfunction. The relationship between these two brain disorders has been debated since the time of Gowers, who referred to migraine as a “Borderland of Epilepsy” in the 20th century [32]. The genetic predisposition and underlying pathophysiological mechanism involving channelopathies are well documented in both of these episodic disorders. Epileptic seizures are also associated with cognitive dysfunction; epilepsy and migraine share similarities in various aspects including epidemiology, pathophysiology and clinical features including postdromal lethargy and a link with sleep that may make the diagnosis challenging.

For careful classification criteria to be established, the term confusion should be discussed. Confusion is a broad term defined as a disturbed mental status. The definition involves an inability for clear and coherent thought and speech [33]. Alterations in thought and speech fall into the category of cognitive disturbances, which can include disorientation and/or deficits in attention, executive function, memory, language, visuospatial ability/perception [34]. Some of the cognitive disturbances described by migraine patients include concentration difficulties, memory complaints, difficulty reasoning and difficulty thinking [35]. Moreover, ACM manifests as a reversible acute confusional state with cardinal findings of agitation and disorientation, speech difficulties and amnesia of the event upon resolution of attack. Broadly speaking, the attacks resemble delirium (also known as acute confusional state, among other terms) which is defined by the Diagnostic and Statistical Manual of Mental Disorders (DSM-5) as an acute change in mental status that affects attention, awareness and cognition [34]. However, the waxing and waning sensorium of delirium is not a typical feature described in ACM.

Patients with most common forms of migraine report attention and cognitive disturbances; however, awareness is generally intact. Episodes with lack of awareness and acute confusional states resembling ACM have also been seen in migraine with brainstem aura, familial hemiplegic migraine (FHM) and other neurological diseases such as episodic ataxia type 2 (EA2) and CADASIL [23,36,37,38,39]. FHM, EA2 and CADASIL have in common genetic abnormalities in chromosome 19 that either affect ion channel function or normal function of vascular smooth muscle cells [40].

### 4.1. Auras

The confusional symptoms may be a manifestation of cortical spreading depression (CSD), the pathophysiological correlate of migraine aura, as it moves across the cortex to higher areas of cortical processing. Moreover, some of the ACM patients described in the literature developed typical auras prior to or during the confusional state. As stated, some of the described symptoms include visual (33%) and/or somatosensory symptoms (19%) and/or speech problems (39%) either prior or during the confusional state. The duration of each of these symptoms was not specified in the reported cases. Some of the visual auras described include “typical visual auras”, unilateral or bilateral blurred vision, and less frequently acute monocular blindness preceded by bright colored lights prior to confusion [2], scintillating scotoma and complete blindness [11]. Some of the speech problems described include transient incoherent speech, aphasia, dysarthria, and slurred speech. Transient expressive or receptive aphasia may also be due to a migrainous phenomenon in addition to other neurological etiologies. The diagnosis can be obtained more readily when attacks are stereotyped and associated with visual phenomenon. The lack of diagnosis may be due to headache being mild, minimized or at times not present.

Prior literature reports of what was previously known as basilar artery migraine has now been modified and replaced with brainstem aura [30]. Migraine with brainstem aura can present with a decrease level of consciousness, which involves lack of awareness, and a decrease level of arousal. Arousal, however, is usually not decreased in ACM; and other brainstem signs and symptoms such as tinnitus, vertigo, diplopia and ataxia typically seen in migraine with brainstem aura are usually absent in ACM. Some of the EEG findings reported in basilar artery migraine [41,42,43,44,45,46,47,48,49] and ACM [1,2,3,4,6,19,24] include FIRDA pattern and slow wave abnormalities in the delta theta range, however unilateral slowing in the delta-theta range has also been described in migraine with aura [45]. The presence of frontal intermittent rhythmic delta activity or FIRDA on EEG is a non-specific finding that can be found in patients with different conditions including toxic or metabolic encephalopathies and brain structural abnormalities [50,51,52].

Mild head trauma is a common trigger in hemiplegic migraine and ACM. In our review of ACM patients, a history of mild head trauma without loss of consciousness prior to the confusional state was present in 33 patients (27%), absent in 50 patients (42%), and was not reported in 37 patients (31%). ACM attacks that followed head injury exhibited clinical features similar to those without any prior trauma. However, a large percentage were teenage boys who complained of recurrent ACM attacks in association with mild head trauma during contact sports and activities. Migraines that occur after a mild head trauma have been called trauma-triggered migraines by some in the literature [15,19,53]. The underlying mechanism is unknown, although in some patients with channelopathies (FHM, EA2), it has been speculated that channel dysfunction could promote a lower threshold for cortical spreading depression leading to brain cellular damage and blood brain barrier disruption after mild head trauma or even stressful situations [40].

### 4.2. Premonitory Symptoms

ACM could conceivably be a part of a premonitory phase, a period of non-headache symptoms that occurs hours to days before the headache begins. Cognitive disturbances, among other non-headache symptoms, have been reported during all migraine phases. Karsan, et al. [54] studied and characterized premonitory symptoms reported in 100 randomly selected children with a final diagnosis of migraine or New Daily Persistent Headache with migrainous features, ages 18 months to 15 years (majority from 5–12 years of age), in whom at least one premonitory symptom had been documented. Fatigue, mood change and neck stiffness were the most common documented premonitory symptoms. Concentration difficulties and memory complaints were documented but seen less commonly. However, this study was not intended to study specifically cognitive symptoms in migraine phases and thus description is limited. Another study evaluating non-headache symptoms in migraine reported tiredness, stiff neck and difficulty concentrating as their most common premonitory symptoms [55].

### 4.3. Headache Phase

Although headache was not reported in all of the cases, the ones that did experienced them either prior or during the confusion. Furthermore, cognitive symptoms are increasingly recognized during migraine attacks. Gil-Gouveia et al. [36], in a cross sectional survey of 165 episodic migraine patients with or without typical aura, ages ranging from 16–63 years, studied the frequency and characteristics of subjective cognitive symptoms during a migraine attack. The most common cognitive complaints were those in executive function category, included difficulty with cognitive processing efficiency/reasoning, difficulty in maintaining attention, and slowed processing speed. In language category, speech fluency was the most common complaint with the majority of patients reporting difficulty talking, not being able to talk or feeling the need to abbreviate conversations. In another prospective study that involved 121 migraine patients that collected non-headache symptoms of migraine, cognitive disturbances including difficulty with concentration, difficulty with thoughts, difficulty reading or writing and difficulty with speech, were reported during all migraine phases, but seen more pronounced during the ictal phase [55]. This same study reported tiredness, difficulty concentrating and stiff neck as their most common postdrome symptoms. The resolution of the symptoms with deep sleep in some of the reported cases is consistent with typical forms of migraine and supports hypothalamic involvement as part of the possible pathological mechanism.

### 4.4. Postdrome Phase

It is also conceivable that ACM could represent the postdrome of migraine. Once headache resolves during the postdrome, patients often describe a sensation of brain “fog,” feeling hungover, and/or confusion. Other studies investigating premonitory and postdrome symptoms in migraine patients report cognitive changes, tiredness and mood changes as part of the overall symptoms, however no detailed description of the cognitive changes is provided [56,57]. Another recent study investigating cognitive dysfunctions and psychological symptoms in migraine without aura excluded patients “exhibiting symptoms compatible with acute confusional migraine during migraine attacks” [58].

Since there are no adult or pediatric controlled studies in the treatment of ACM, evidence-based guidelines are unavailable. Based on the review, we found migraine treatments were often used. Migraine specific treatments such as triptans and ergotamine or nonspecific treatments such as dopaminergic antagonist [21] and over-the-counter pain medications were often used. Common preventive medications such as anti-hypertensive (propranolol) [10,28], and anti-seizure (topiramate, valproic acid) [11,19,22,24,27] were used. These medications might be effective in the prophylaxis of ACM due to inherent ability to affect CSD [59] as well as modulate altered neurotransmission of the trigeminovascular pathway [60,61].

ACM, although rare, is an important migraine variant seen in ambulatory neurology practice. Because of the paucity of related literature and lack of distinctive classification by ICHD-III β, most clinicians are not well aware of it. The agitation associated with it might prompt an emergency visit, and the early recognition of this disorder is important to avoid unnecessary and invasive diagnostic procedures. Proposed criteria for ACM has been provided by Pacheva et al. and Schipper et al. [25,62]. Based on the clinical data available so far, we are generally in agreement with the proposed criteria with the exception of some modifications that can serve as basis for the inclusion and classification in the Appendix section under A1.6: episodic syndromes that may be associated with migraine. The hallmark of this variant is sudden development of confusional state which is varied in presentation, from inattention to spatial disorientation. We made an effort to define this reversible altered mental status in detail. The age limit cannot be specified in the criteria as a few of these cases, i.e., approximately 17%, are reported in the adult population apart from children and early adolescents [6,20,23,27,29]. Moreover, the reference to pediatric syndromes may result in missed diagnoses especially in the elderly population [31]. The confusion, in most instances, is accompanied by behavioral, language and memory disturbances. Patient might experience visual, sensory aura and headache with migrainous character and features, however its onset is not specified. The attack lasts from few minutes to many hours, but no more than 24 h from the time of onset. Sleep can help in the resolution of attacks as the patient wakes up symptom free with amnesia for the whole episode. Mild head trauma can act as a trigger in both trauma triggered migraine and ACM, and the altered consciousness following the head injury may lead to the erroneous diagnoses of concussion and intracerebral hemorrhage. It is our impression that ACM related to traumatic brain injury (TBI) should be viewed with caution as many of the cases occurred close in timing to the mild head injury and could therefore be difficult to separate from a secondary headache type such as post-concussive headache. Therefore, a history of TBI was not included as criteria for ACM.

A past medical and family history of migraine should be taken into consideration while making a definitive diagnosis. The relation of trauma with the onset of ACM attack needs to be explored further. No significant abnormalities in routine laboratory and imaging tests are noted, except slowing and FIRDA pattern EEG, which resolve over a period of time. Based on these clinical findings and the available literature, the proposed classification criteria is listed in Table 2.

## 5. Conclusions

ACM is a migraine variant that is not well understood. While it is true that ACM can be recognized by its unique characteristics, some of its symptoms and signs overlap with non-headache features of migraine with and without aura. The lack of specific classification criteria may lead to diagnostic uncertainty, excessive diagnostic testing and delays in adequate treatment. Prospective studies are needed to further study this disorder and its association with other migraine forms. Inclusion in the appendix of ICHD-3β criteria is necessary for better characterization through research efforts.

## Figures and Tables

**Table 1:** Brief description of previously reported cases of acute confusional migraine including demographic information of the patients (age, gender), clinical presentation (predominant symptoms and percentage of people exhibiting that symptom, onset of headache relative to confusion, duration of confusional state), treatment, and routine follow up if any (recurrence and outcomes) *.

Case Report (Reference Number)	No. of Patients (*n*)	Gender Ratio (M:F)	Age Range (Years)	Mean Age (Mean ± SD)	Clinical Presentation ^α^	Headache Onset Pre/Post-Confusion	Duration of Confusion (h)	Treatment	Recurrence	Outcomes
Gascon G. and Barlow C. [1]	4	3:1	8–16	12.3 ± 3.5	Disorientation (100%), Agitation (100%), Speech (100%), somatosensory (25%) and memory disturbances (50%)	Pre-confusion (4)	4–24 h	Ergotamine and Phenobarbital	None	Two patients had multiple episodes of headache afterwards
Emery III et al. [3]	4	3:1	5–14	10 ± 4.2	Confusion (100%), agitation (100%), visual (50%), somatosensory (50%) speech (25%), and memory disturbances (75%)	Pre-confusion (4)	1.5–9 h	NA	Two patients reported similar episodes in the past	Three patients had intermittent episodes of headache
Ehayi A. and Fenichel G. M. [2]	5	3:2	9–14	11 ± 2	Confusion and disorientation (100%), Agitation (100%), visual (80%), somatosensory (20%), speech (40%), motor (20%) and memory disturbances (80%)	Pre- (4), post-confusion (1)	0.5–24 h	Ergotamine, methysergide	Over brief period of time all patients had recurrent ACM episodes	Migraine
Parrino L. et al. [4]	2	2:0	14–15	14.5 ± 0.7	Confusion and disorientation (100%), agitation (100%), Photophobia (50%), visual (100%), somatosensory (50%), speech (100%), and memory disturbances (100%)	Pre-confusion (2)	24 h	NA	One patient reported similar episode in the past	None
Sacquegna T. et al. ^1^ [5]	1	0:1	17	17 ± 0	Confusion and disorientation (100%), visual (100%), somatosensory (100%), and memory disturbances (100%)	Pre-confusion (1)	2 h	NA	Several episodes marked by less confusion	NA
Pietrini V. et al. [6]	12	6:6	8–60	19.4 ± 13.4	Confusion (100%), agitation (100%), visual (42%), somatosensory (42%), speech (25%), and motor symptoms (17%)	Pre-confusion (10)	1–12 h	NA	NA	NA
Haan J. et al. [7]	1	0:1	13	13 ± 0	Confusion and disorientation (100%), agitation (100%), memory disturbance (100%)	Pre-confusion (1)	12 h	NA	One similar episode in the past	NA
Piatella L. et al. [8]	5	4:1	10–16	12.6 ± 2.3	Confusion and disorientation (%100), agitation (20%), speech (80%), somatosensory disturbances (20%)	Pre-confusion (4) ^2^	15 min–24 h	NA	NA	Three patients developed migraine
D’Cruz O. and Walsh D. J. [9]	3	0:3	11	11 ± 0	Confusion and disorientation (100%), speech (67%), visual (67%), memory disturbances (100%)	Pre-confusion (3)	6 h	NA	NA	NA
Sheth R. D. et al. [10]	6	1:5	7.5–17	11.8 ± 3.5	Confusion and disorientation (100%), agitation (100%), photophobia (50%), visual (50%), memory disturbances (100%)	NA ^2^	1–12 h	Propranolol	Two patients had recurrent ACM episodes	NA
Ferrera P. and Reicho P [11]	2	1:1	6–9	7.5 ± 2.1	Confusion (100%), agitation (50%), visual (50%), somatosensory (100%), speech (50%), and motor disturbances (50%)	Pre-confusion (1) ^2^	NA	Sodium Valproate	Both patients had episodes of confusion in past	One patient had 2 episodes of headache
Shaabat A. et al. [12]	13	11:2	6–15	10.8 ± 2.9	Confusion (100%), agitation (62%)	Pre-confusion (13)	1.5–24 h	NA	Four patients had recurrent ACM episodes	NA
Nezu A. et al. [13]	2	1:1	7–12	9.5 ± 3.5	Confusion (100%), Photophobia (50%), visual (50%), somatosensory (50%), speech (50%), motor (50%) and memory disturbances (100%)	Post-confusion (2)	6–12 h	Dihydergot	NA	NA
Neinstein L. and Milgrom E. [14]	1	1:0	14	14 ± 0	Confusion (100%), anisocoria (100%), and ataxic gait (100%)	Pre-confusion (1)	NA	High-dose Oxygen and Sumatriptan	One similar episode	NA
Soriani S. et al. [15]	11	8:3	6–14	9 ± 3	Confusion (100%), agitation (45%), somnolence (55%), visual (27%), speech disturbances (9%)	Six patients had headache ^3^	1–12 h	NA	NA	Four patients developed migraine with aura & one w/o aura
Al-Twaijri W. and Shevell M. [16]	5	2:3	6.5–15	10.9	Confused, agitated and memory disturbances ^4^	NA ^2^	NA	NA	NA	NA
Bechtel K. et al. [17]	2	1:1	11–14	12.5 ± 2.1	Confusion (100%), speech (100%), visual (50%), somatosensory (50%) and memory disturbances (50%)	Pre- (1), post confusion (1)	Several hours	Acetaminophen	None	One patient had several episodes of headache
Gascon G. G. et al. [18]	13	6:7	6–16	12.3 ± 3.8	Confusion (69%), speech (46%), somatosensory (7.7%) and memory disturbances (8%)	Pre- (7), post confusion (4) ^3^	NA	NA	Two patients had recurrent episodes of ACM	None
Fujita M. et al. [19]	1	0:1	10	10 ± 0	Confusion and disorientation (100%) visual disturbances (100%)	Pre-confusion (1)	5–10 h	Sodium Valproate	Recurrent ACM episodes	Attacks were controlled after increasing the dose of sodium valproate
Sathe S. et al. [20]	7	5:2	42–58	51.9 ± 7.3	Confusion (100%), agitation (100%), visual (100%), somatosensory (57%), speech (57%), motor (14%) and memory disturbances (100%)	NA ^2^	NA	NA	Recurrent ACM episodes	CADASIL
Khatri et al. [21]	2	1:1	11–16	13.5 ± 3.5	Confusion (100%), speech (50%) and memory disturbances (50%)	Pre-confusion (2)	0.5–72 h	Prochlorperazine	Recurrent ACM episodes	Prochlorperazine was effective in acute management
Avraham S. B. et al. [22]	1	1:0	12	12 ± 0	Confusion (100%), speech (100%), visual (100%) and somatosensory symptoms (100%)	Pre- (3), during (5), post-confusion (1)	NA	Sodium Valproate	None	None
Gantebein A. et al. [23]	10	6:4	16–62	30.5 ± 14.7	Confusion (100%), agitation (20%), photophobia (10%), visual (40%), somatosensory (10%), motor (10%) speech (40%), and memory disturbances (60%)	Pre- (4), post-confusion (3) ^2^	1–6 h	NA	Seven patients had recurrent confusional episodes	NA
Rota E. et al [24]	1	0:1	12	12 ± 0	Confusion (100%), agitation (100%)	Not specified ^2^	4 h	Topiramate prophylaxis	Previous attack characterized by less agitation	No further episodes of confusion and headache after topiramate
Pacheva I. and Ivanov I. [25,26]	3	1:2	12–14	12.7 ± 1.2	Confusion (100%), agitation (67%), visual (33%), somatosensory (33%), motor (33%), speech (100%) and memory disturbances (67%)	Pre-confusion (3)	8–10 h	Diazepam and phenobarbital	None	Two patients had 1–2 episodes of migraine without aura per month
Verma R. et al. [27]	1	0:1	29	29 ± 0	Confusion (100%), Agitation (100%), and memory disturbances (100%)	Pre-confusion (1)	NA	Sodium Valproate	None	NA
Kim D. et al. [28]	1	0:1	9	9 ± 0	Confusion (100%), agitation (100%), speech (100%) and memory disturbance (100%)	NA ^2^	2 h	Propranolol & Flunarizine	Similar episode in the past	Migraine
Sato K. et al [29]	1	1:0	24	24 ± 0	Confusion (100%), agitation (100%), visual (100%) and speech disturbance (100%)	Pre-confusion (1)	NA	Propofol	None	NA
	Total (*n* = 120)	68:52	5–62 years		Confusion and disorientation (100%), agitation (53%), photophobia (5%), visual (33%), somatosensory (19%), motor (6.7%), speech (39%), memory disturbances (39%)	Pre- (69), post-confusion (11)	15 min–72 h	----------	----------	-----------

^1^ The patient had not been labeled as having ACM although the clinical and Electroencephalography (EEG) findings were similar to those seen in ACM; ^2^ All/some, patients developed headache but its onset relative to confusion and disorientation was not specified; ^3^ Not all patients in respective case reports and series complained of headache during ACM episode; ^4^ Information about percentage of people exhibiting that symptom is not specified; * Adapted and modified from previous articles; NA: not available in the particular article; ^α^ Sensory symptoms are described as visual and somatosensory symptoms.

**Table 2:** Proposed Classification Criteria for A1.6 Episodic Syndromes that may be associated with migraine: Acute Confusional Migraine.

(A) At least one attack, fulfilling criteria B to G, not attributed to other medical disorder and/or drug intoxication:
(B) At least one of the following:
Decreased attention Altered awareness Impaired cognition (disorientation and/or deficits in attention, executive function, memory)
(C) At least one of the following:
Agitation or combative behavior Perception disturbances (i.e., visuospatial abnormalities, photophobia) Slowing or frontal intermittent rhythmic delta activity on EEG with complete resolution within a week Aura (reversible visual, sensory, language or brainstem disturbance) for <1 h (typical)
(D) Complete resolution within 24 h or after sleep with partial or complete amnesia of event
(E) Normal neurological or no persistent neurologic deficit examination following the attack
(F) At least one of the following:
Past medical history of migraine Family history of migraine Headache, if present, may occur before, during and after the confusional state
(G) Not attributed to another disorder

## Abbreviations

ACM	Acute confusional migraine
ICHD-3β	International Headache Society’s International Classification of Headache Disoders-3 beta version
FHM	Familial hemiplegic migraine
EA2	Episodic ataxia type 2
CADASIL	Cerebral Autosomal-Dominant Arteriopathy with Subcortical Infarcts and Leukoencephalopathy
MRA	Magnetic resonance imaging
FIRDA	Frontal intermittent rhythmic delta activity
CSD	Cortical Spreading Depression
EEG	Electroencephalography
